# Interleukin-17 Receptor D in Physiology, Inflammation and Cancer

**DOI:** 10.3389/fonc.2021.656004

**Published:** 2021-03-23

**Authors:** Charlotte Girondel, Sylvain Meloche

**Affiliations:** ^1^Institute for Research in Immunology and Cancer, Montreal, QC, Canada; ^2^Department of Pharmacology and Physiology, Faculty of Medicine, Université de Montréal, Montreal, QC, Canada; ^3^Molecular Biology Program, Université de Montréal, Montreal, QC, Canada

**Keywords:** IL-17RD, cancer, tumor suppressor, MAPK signaling, inflammation

## Abstract

Interleukin-17 receptor D (IL-17RD) is an evolutionarily conserved member of the IL-17 receptor family. Originally identified as a negative regulator of fibroblast growth factor (FGF) signaling under the name of Sef (Similar expression to FGF genes), IL-17RD was subsequently reported to regulate other receptor tyrosine kinase signaling pathways. In addition, recent studies have shown that IL-17RD also modulates IL-17 and Toll-like receptor (TLR) signaling. Combined genetic and cell biology studies have implicated IL-17RD in the control of cell proliferation and differentiation, cell survival, lineage specification, and inflammation. Accumulating evidence also suggest a role for IL-17RD in tumorigenesis. Expression of IL-17RD is down-regulated in various human cancers and recent work has shown that loss of IL-17RD promotes tumor formation in mice. However, the exact mechanisms underlying the tumor suppressor function of IL-17RD remain unclear and some studies have proposed that IL-17RD may exert pro-tumorigenic effects in certain contexts. Here, we provide an overview of the signaling functions of IL-17RD and review the evidence for its involvement in cancer.

## Introduction

Interleukin-17 (IL-17) A and IL-17F cytokines, which are members of the IL-17 family along with IL-17B, IL-17C, IL-17D and IL-17E/IL-25, play a central role in host defense against bacterial and fungal infections as well as in tissue repair (1, 2). However, when chronically activated, these cytokines contribute to inflammatory disorders such as psoriasis and arthritis. Accumulating evidence also implicates IL-17A in the initiation and progression of inflammation-associated cancer, notably colitis-associated cancer (1–4). IL-17A and IL-17F are signature cytokines of T helper 17 (Th17) cells, but they are also produced by other innate immune cells in peripheral tissues and by some epithelial cells (2, 5). The functions of other IL-17 family members are less well characterized.

IL-17 cytokines signal through members of the IL-17 receptor family, which comprises the five subunits IL-17RA, IL-17RB, IL-17RC, IL-17RD and IL-17 RE (6). The prototypical IL-17A and IL-17F bind to the heterodimeric complex formed by IL-17RA and IL-17RC. Much remains to be learned about the roles of the other IL-17 receptors. IL-17RD, also known as Sef for Similar expression to fibroblast growth factor (FGF) genes, was originally described as an antagonist of the FGF receptor signaling pathway (7, 8). Subsequent studies suggested that IL-17RD functions as a signaling hub that interacts with components of the ERK1/2 mitogen-activated protein kinase (MAPK) cascade and with innate immune signaling pathways (9–11). Thus, IL-17RD has been implicated in a variety of cellular responses such as cell proliferation, differentiation, survival, migration and invasion, and inflammation. Genetic studies have revealed that mice with a disruption of *Il17rd* gene are viable but show mild defect in auditory brainstem responses and increased cortical bone mass (12–14). However, the long-term impact of IL-17RD deficiency or its role in inflammation and tumorigenesis were not addressed in these studies.

Cellular studies have suggested a role for IL-17RD in tumorigenesis, although the mechanisms involved have remained unclear and debatable. More recent findings have now provided compelling evidence for a tumor suppressor function of IL-17RD and started to shed light on the underlying biology. In this review, we first summarize the biological activities of IL-17RD. Then, we discuss the role of IL-17RD in tumor development and progression, and highlight some of the remaining questions and discrepancies in the field.

## IL-17RD is a Regulator of Receptor Tyrosine Kinase and Innate Immune Signaling

### IL-17RD Negatively Regulates Receptor Tyrosine Kinase-Dependent Signaling Pathways

IL-17RD was first identified as a feedback inhibitor of the FGF-dependent ERK1/2 MAPK signaling pathway in zebrafish (7, 8). Subsequent studies in mammalian cells confirmed the antagonistic action of IL-17RD on FGF receptor signaling and further documented its modulatory effect on other receptor tyrosine kinases (RTKs) (15–20). Indeed, bone marrow cells from IL-17RD-deficient mice show enhanced ERK1/2 activation by FGF2, whereas cells derived from IL-17RD transgenic mice show decreased FGF2 signaling (13). The exact mechanism by which IL-17RD antagonizes ERK1/2 signaling is controversial as different studies have proposed that IL-17RD directly interferes with the FGF receptor itself (8, 16, 19), or acts downstream at the level of RAS (21) or MEK1/2 (7, 17, 18, 22). Contrary to these studies, other groups reported that IL-17RD overexpression potentiates epidermal growth factor (EGF)-stimulated ERK1/2 activation in 293 cells (23) and steady-state ERK1/2 phosphorylation in mesenchymal lung cancer cells (24). Although most studies have focused on the regulation of the ERK1/2 MAPK pathway, expression of IL-17RD was also reported to inhibit FGF-stimulated AKT activation in fibroblasts (25). Other studies showed that IL-17RD when overexpressed in 293 cells can physically interact with TAK1 and mediate JNK and p38 MAPK activation, resulting in apoptosis (26, 27). It has been suggested that the modulatory effects of IL-17RD on RTK signaling are dependent on isoform type and cellular context (11). Differences in experimental conditions are also likely to explain some of the discrepant findings.

RTKs are critical regulators of cellular processes such as cell proliferation and differentiation, survival, metabolism, migration, and angiogenesis (28, 29). In agreement with its role as a negative regulator of ERK1/2 signaling, overexpression of IL-17RD was consistently found to inhibit RTK-induced proliferation of various immortalized and transformed cell lines *in vitro* (16–18, 22, 25, 30–33). Reciprocally, RNAi silencing of IL-17RD was shown to increase FGF-stimulated HeLa cell proliferation (25, 33). On the other hand, two contradictory studies suggested that IL-17RD expression promotes cancer cell proliferation (24, 34). Ectopic expression of IL-17RD was shown to inhibit FGF-induced PC-12 cell differentiation but to increase EGF effect (19). IL-17RD overexpression also promotes apoptotic cell death (15, 25–27). Consistent with these studies, genetic disruption of *Il17rd* stimulated the expansion of cultured osteoblast progenitors by increasing proliferation and decreasing apoptosis, and enhanced osteoblast differentiation (13).

### IL-17RD Regulates Inflammation Signaling Pathways

The role of IL-17RD in mediating signaling by IL-17 cytokines has remained controversial. Rong et al. (35) first reported that IL-17RD associates with IL-17RA and TRAF6 when overexpressed in cells, and promotes basal and IL-17A-induced gene expression. A more recent study confirmed that IL-17RD heterodimerizes with IL-17RA and proposed that IL-17RD serves as an alternative functional receptor for IL-17A in keratinocytes (36). In this model, IL-17RD mediates IL-17A-induced pro-inflammatory gene expression in keratinocytes and psoriasis-like skin inflammation. On the other hand, Mellett et al. (10) reported that IL-17RD differentially regulates various IL-17A-responsive gene programs. Loss of *Il17rd* was shown to inhibit IL-17A-stimulated neutrophilia in mice, concomitant with reduced p38 MAPK activation and MIP-2 expression, while enhancing IL-17A-induced NF-kB activation and expression of IL-6 and KC genes. It has been suggested that functional compensation of IL-17RD and IL-17RC, the two heterodimeric partners of IL-17RA, may explain the contradictory functions of IL-17RD on IL-17A signaling (36). IL-17A is the only ligand of IL-17RD identified to date.

IL-17RD was also described as a negative regulator of cytokine and Toll-like receptor (TLR) signaling. It was reported that expression of IL-17RD inhibits IL-1 and TNFα-induced NF-kB activation by sequestering the NF-kB subunit p50 in the cytoplasm, thereby attenuating IL-6 gene expression (9). Another study showed that IL-17RD deficiency enhances TLR-dependent signaling and pro-inflammatory gene expression, and increases the susceptibility of mice to TLR-induced septic shock (37). The authors determined that the SEFIR domain of IL-17RD interacts with the TIR domain of the adaptor protein MyD88 to block TLR downstream signaling. In a recent study, we provided additional evidence that IL-17RD negatively regulates TLR as well as IL-17A signaling in epithelial cells (38). Importantly, we found that genetic inactivation of *Il17rd* markedly exacerbates the inflammatory response in a model of colitis-associated cancer, illustrated by a strong enrichment in inflammation-related gene signatures in colon tumors and elevated expression of pro-inflammatory cytokines (38). All these studies identify IL-17RD as a key regulator of inflammation signaling pathways.

## Role of IL-17RD in Tumorigenesis

**Figure 1:**
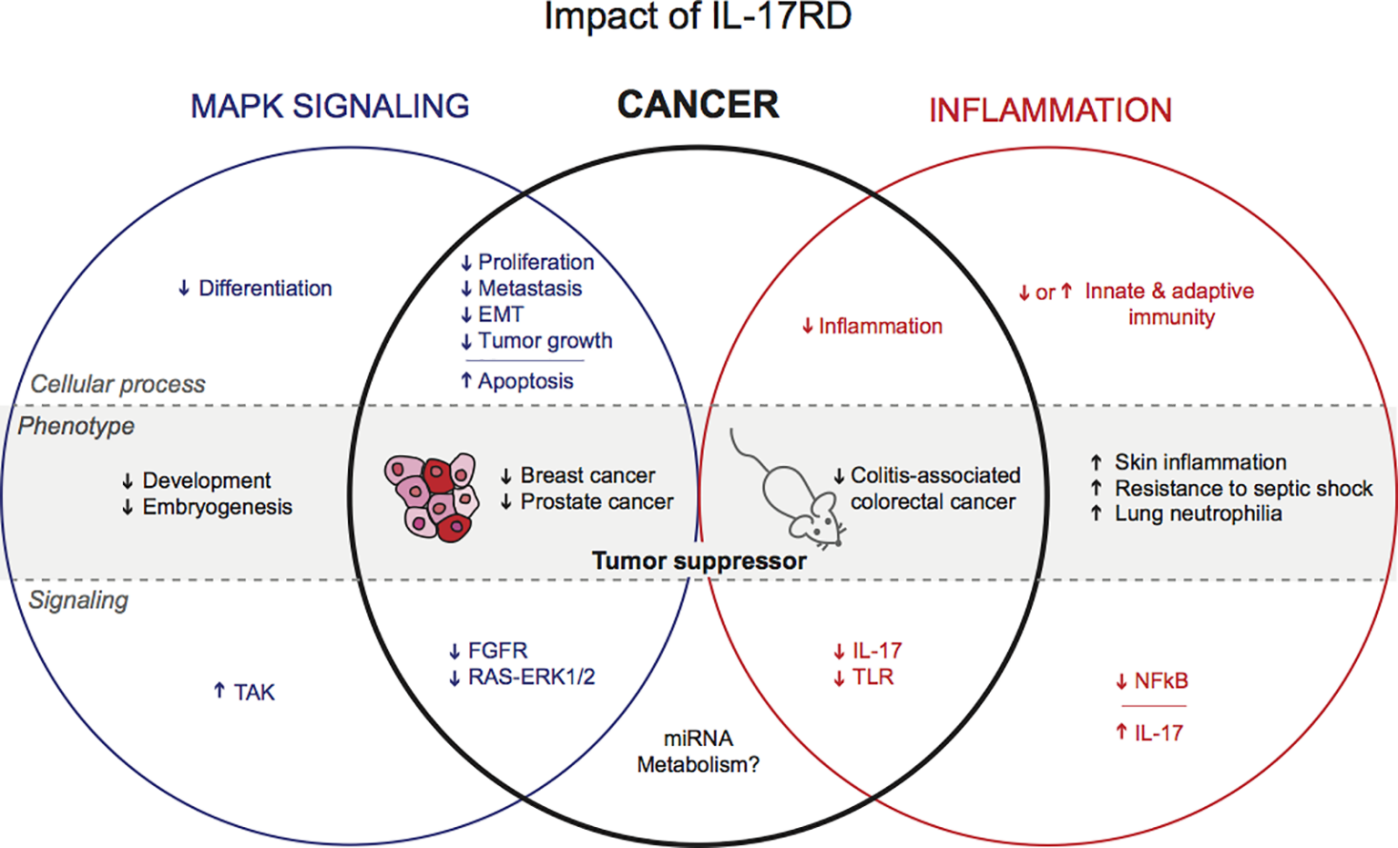
Postulated functions of IL-17RD in physiology and cancer.

The negative regulatory function of IL-17RD in RTK mitogenic signaling and inflammation-related signaling pathways suggest a possible involvement of IL-17RD in tumorigenesis (**Figure 1**). Hyperactivation of RTK signaling by receptor overexpression or mutational activation is a frequent alteration in human cancer (39). Chronic inflammation is also a common feature of many tumors and is now considered as a hallmark of cancer (40). However, until recently, the role of IL-17RD in tumor biology had not been studied *in vivo*. Moreover, the relative importance of IL-17RD-modulated signaling events in tumorigenesis has remained elusive.

### IL-17RD Is Down-Regulated in a Variety of Human Cancers

A number of studies have examined the expression of IL-17RD in clinical samples. Darby et al. (41) showed that IL-17RD mRNA and protein expression are down-regulated in advanced prostate cancer, negatively correlating with Gleason score and metastatic dissemination. In a follow-up study on a larger clinical cohort of prostate cancers, the authors reported that IL-17RD is strongly expressed in 74% (32/43 cases) of benign tissues, but in only 21% (37/176 cases) of cancer biopsies. IL-17RD protein was weak or absent in 46% (17/37 cases) of patients with bone metastasis and in 17% (18/104 cases) of men without metastasis (30). Another study showed that IL-17RD mRNA is down-regulated in a variety of carcinomas, including breast, ovary, thyroid and prostate. IL-17RD loss was significantly correlated with tumor progression (33). Interrogation of the TCGA and GTEx databases also revealed that expression of IL-17RD mRNA is lower in colon adenocarcinomas as compared to adjacent tissue and normal intestinal mucosa. *IL17RD* is significantly downregulated in the microsatellite instability subgroup of colorectal cancer patients (38). These observations are consistent with the hypothesis of a tumor suppressor function of IL-17RD.

### IL-17RD Is a *Bona fide* Tumor Suppressor in Mice

*In vitro* studies have shown that IL-17RD exerts inhibitory effects on RTK-induced ERK1/2 MAPK signaling, and possibly other downstream signaling pathways, to restrain the proliferation of various cancer cell lines (22, 25, 30, 32, 33). High expression of IL-17RD also promotes apoptosis of 293 cells (26, 27). In addition to its effect on cell proliferation and survival, several studies have documented the impact of IL-17RD expression on other tumorigenic properties. Ectopic expression of IL-17RD in RAS-transformed intestinal epithelial cells was shown to reverse the morphologically transformed phenotype and to prevent RAS-mediated polyploidization of the cells (22). IL-17RD expression also restrains the motility and invasion of prostate and breast cancer cells (30, 41, 42). The effect of IL-17RD has been associated with down-regulation of the metalloprotease MMP9 (41) and with reduced expression of epithelial-mesenchymal transition (EMT) markers such as Snail and Slug (42). Importantly, overexpression of IL-17RD was found to inhibit tumor growth in mouse xenograft models of prostate, intestinal and breast cancer (22, 30, 42, 43). IL-17RD also decreased metastatic incidence of xenografted PC3M prostate cancer cells, consistent with clinical observations (43). All these findings point toward a possible tumor suppressor role of IL-17RD.

To directly address the role of endogenous IL-17RD in tumorigenesis, we have analyzed a longitudinal cohort of wild type, heterozygous and homozygous *Il17rd* mutant mice. We found that deficiency of IL-17RD leads to the formation of spontaneous tumors in aging mice in a gene dosage-dependent manner (38). Histological analysis revealed the presence of tumors in the lungs, female reproductive organs, lymphoid organs and gastrointestinal tract. We further showed that loss of IL-17RD promotes the development of intestinal tumors in the azoxymethane-dextran sodium sulfate (AOM-DSS) model of colitis-associated colorectal cancer. This study provided the first demonstration that *Il17rd* is a *bona fide* tumor suppressor gene in mice.

### IL-17RD Limits Tumor-Associated Inflammation Without Affecting Cell Proliferation

**Figure 2:**
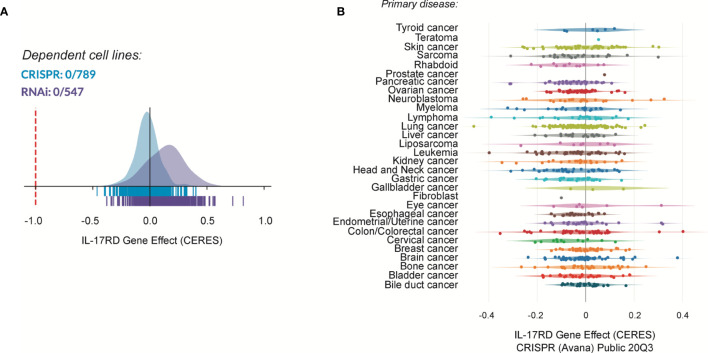
Cancer dependency map (depmap) analysis of IL-17RD. **(A)** Cancer cell line dependency of *IL17RD* gene from CRISPR/Cas9 and RNAi datasets. **(B)** Dependency map of *IL17RD* across different cancer histological types. CERES is a computational method to estimate gene dependency scores from CRISPR/Cas9 and RNA interference essentiality screens. A lower score indicates a higher probability that the gene is essential in a given cell line. A score of 0 indicates a gene that is not essential whereas a score of -1 corresponds to the median of all essential genes (red line).

IL-17RD may suppress tumor development and progression by multiple mechanisms. Given the multiple *in vitro* studies documenting the antagonistic effect of IL-17RD on mitogenic signaling pathways and cell proliferation, it is tempting to speculate that the protein may exert its tumor suppressor function by curtailing cancer cell proliferation. Surprisingly, we did not observe any difference in the number of phospho-ERK1/2 and Ki-67 positive cells between wild type and *Il17rd^-/-^* colon tumors in the AOM-DSS model (38). Similarly, we failed to detect changes in ERK1/2 phosphorylation and cell proliferation in the intestinal epithelium of *Il17rd^-/-^* mice as compared to control animals. We also found that depletion of IL-17RD by siRNAs did not affect the proliferation rate of human colon carcinoma HCT 116 cells and modestly reduced proliferation of murine MC-38 cells. Thus, we concluded that IL-17RD deficiency alone has no significant impact on the proliferation of normal or transformed intestinal cells (38). Interestingly, systematic analysis of IL-17RD dependency using the publicly available depmap portal (https://depmap.org/portal/depmap/) revealed that no single cancer cell line shows a dependency on IL-17RD expression for their proliferation (**Figure 2**). In fact, depletion of IL-17RD by CRISPR/Cas9 gene editing or by RNA interference had no significant effect on the proliferation or survival of more than 700 cancer cell lines of different histological origins. There are many possible explanations for these discrepancies about the role of IL-17RD in cancer cell proliferation. First, the loss of IL-17RD might be compensated for by other regulators of the ERK1/2 MAPK pathway, for example Sprouty proteins, or by a rewiring of mitogenic signaling pathways. This would also explain the mild phenotype of IL-17RD-deficient mice. Second, the negative regulatory effect of IL-17RD might be specific to FGF signaling and/or to the cellular type or context. Third, variations in experimental conditions, such as differences between gain-of-function and loss-of-function studies, or technical issues such as off-target effects of RNAi reagents. Additional studies with genetically-engineered mouse models and CRISPR/Cas9 edited cells should help clarify these questions.

Inflammation can play a good or bad role in the tumorigenic process. An increased innate immune response as a result of cancer-associated genetic alterations or tissue damage can trigger anti-tumor immunity and prevent tumor formation. In contrast, chronic inflammation promotes tumor development and has been shown to play a critical role in the etiology of many cancers (44). In the AOM-DSS model, we observed that *Il17rd^-/-^* mice display an exacerbated inflammatory response characterized by a higher colitis score, increased immune cell infiltration in colonic tissue, and an increase in circulating pro-inflammatory cytokines (38). Colon tumors from *Il17rd^-/-^* mice showed elevated expression of pro-inflammatory cytokines including IL-17A and IL-6. Notably, previous studies have shown that IL-17RD negatively regulates signaling by IL-1/TNFα, IL-17A and TLRs in other cellular contexts, resulting in decreased expression of IL-6 and other pro-inflammatory cytokines and chemokines (9, 10, 37). IL-6 and IL-17A are known to play a key role in the promotion and progression of colorectal cancer and other inflammation-associated solid tumors (45, 46). Thus, downregulation of IL-17RD expression may favor the creation of an inflammatory tumor microenvironment conducive to tumor development. Interestingly, we also observed that expression of pro-inflammatory cytokine genes is up-regulated in the intestinal tissue of aging *Il17rd^-/-^* mice, which are prone to various cancers (38). These results have led to the hypothesis that IL-17RD supresses tumorigenesis by limiting the extent and duration of inflammation.

### Does IL-17RD Exert Pro-Tumorigenic Effects?

Although our recent work has provided compelling evidence for a tumor suppressor function of IL-17RD in mice, other studies have rather suggested that IL-7RD can exert pro-tumorigenic actions in certain contexts. Notably, IL-17RD was found to be the target of several non-coding RNAs in cancer cells. Non-coding RNAs play a major role in the post-transcriptional regulation of gene expression and are often dysregulated in cancer (47). An analysis of microRNA expression profiles in tissues from patients with ulcerative colitis without or with neoplasia identified miR-193a-3p as a miRNA that is significantly down-regulated in ulcerative colitis-associated neoplasia (34). IL-17RD, which is a predicted target of miR-193a-3p, was found to be up-regulated in colitis-associated cancer tissue as compared to normal controls. The authors showed that intraperitoneal injection of miR-193a-3p modestly decreased the growth of xenografted HCT 116 tumor cells expressing wild type IL-17RD as compared to tumors expressing IL-17RD mutated in the 3’UTR, and concluded that miR-193a-3p loss promotes carcinogenesis through up-regulation of IL-17RD. Another study showed that the long non-coding RNA (lncRNA) NEAT1, which competes with miR-193a-3p, is up-regulated in colorectal cancer samples and correlates with poor outcome (48). Depletion of NEAT1 by shRNA in LOVO and HCT 116 colorectal cancer cell lines downregulated IL-17RD mRNA expression, and decreased the tumor growth rate of LOVO xenografts. Two recent studies by the same group reported that the circular RNA circ-PITX1 is up-regulated in glioblastoma tumors and elevates IL-17RD expression by sponging miR-518a-5p (49, 50). Ectopic expression of circ-PITX1 was shown to modestly increase the proliferation, migration and invasion of glioblastoma cell lines *in vitro*. In a similar study, the microRNA miR-506 was reported to inhibit the proliferation and invasion of papillary thyroid carcinoma cells *via* down-regulation of IL-17RD (51). However, all these studies should be interpreted with caution as non-coding RNAs have multiple targets and it is difficult to isolate the impact of a single target mRNA. Intriguingly, a recent study reported that shRNA-mediated depletion of IL-17RD in the mouse epithelial-like lung carcinoma cell line 393P, which modestly slows down proliferation, markedly reduces *in vivo* tumor growth and sensitizes tumors to a MEK1/2 inhibitor (24).

## Conclusions and Perspectives

IL-17RD is a less-well characterized member of the IL-17 receptor family. Genetic and cell biology studies have revealed that IL-17RD acts as a feedback inhibitor of the FGF signaling pathway in different organisms. The phenotype of IL-17RD-deficient mice in development is consistent with such a role. Various studies also suggested that IL-17RD negatively regulates signaling by other RTKs, but the evidence is less compelling. In line with its inhibitory effect on RTK mitogenic signaling, many groups have shown that modulating the expression of IL-17RD impacts on cell proliferation *in vitro*. However, systematic analysis of a large panel of human cancer cell lines failed to show any dependency on *IL17RD* gene, arguing against a cell-autonomous role of the protein in cell proliferation. IL-17RD may regulate proliferation of specific cell lineages in a context-dependent manner during development, but it is unlikely to act as a universal regulator of mitogenic signaling. On the other hand, IL-17 was shown to play a key role in the regulation of innate immune signaling pathways, although much remains to be learned about the molecular mechanisms involved.

Several studies point toward a role of IL-17RD in cancer. Our group has recently reported that loss of *Il17rd* leads to increased tumor formation in aging mice and promotes colitis-associated colon cancer. However, other studies have suggested that IL-17RD exerts pro-tumorigenic effects after *ex vivo* modulation of IL-17RD levels or small non-coding RNAs. The pathophysiological relevance of these studies needs to be confirmed in suitable animal models. Additional studies are required to define the exact mechanisms underlying the tumor suppressor function of IL-17RD in colon cancer and other cancers (**Figure 1**). Among many outstanding questions. What is the relative impact of IL-17RD on RTK and innate immune signaling pathways? What are the direct partners and targets of IL-17RD? How tumor epithelial cells and the tumor microenvironment contribute to tumor suppression by IL-17RD? Interestingly, a recent study has shown that direct ultrasound delivery of IL-17RD gene in mouse prostate tumors reduces tumor cell proliferation, angiogenesis, and tumor growth (52). The clinical significance of IL-17RD as a diagnostic or prognostic marker or as future therapeutic target requires additional investigations.

## Author Contributions

CG and SM wrote the manuscript. All authors contributed to the article and approved the submitted version.

## Funding

Work in SM laboratory was supported by grants from the Cancer Research Society.

## Conflict of Interest

The authors declare that the research was conducted in the absence of any commercial or financial relationships that could be construed as a potential conflict of interest.
